# H-NS Family Proteins Drastically Change Their Targets in Response to the Horizontal Transfer of the Catabolic Plasmid pCAR1

**DOI:** 10.3389/fmicb.2020.01099

**Published:** 2020-05-29

**Authors:** Taisuke Nakamura, Chiho Suzuki-Minakuchi, Hibiki Kawano, Yu Kanesaki, Shinji Kawasaki, Kazunori Okada, Hideaki Nojiri

**Affiliations:** ^1^Biotechnology Research Center, The University of Tokyo, Tokyo, Japan; ^2^Collaborative Research Institute for Innovative Microbiology, The University of Tokyo, Tokyo, Japan; ^3^NODAI Genome Research Center, Tokyo University of Agriculture, Tokyo, Japan; ^4^Department of Molecular Microbiology, Tokyo University of Agriculture, Tokyo, Japan

**Keywords:** nucleoid-associated proteins, H-NS, MvaT, *Pseudomonas*, plasmid, transcriptome

## Abstract

H-NS family proteins regulate the expression of many genes by preferably binding to AT-rich genomic regions and altering DNA topology. They are found in both bacterial chromosomes and plasmids, and plasmid-encoded H-NS family proteins have sometimes been suggested to act as a molecular backup of the chromosomally encoded ones. Pmr is an H-NS family protein encoded on the catabolic plasmid pCAR1, which belongs to incompatibility P-7 group. We have investigated the function of Pmr in *Pseudomonas putida* KT2440, where two H-NS family proteins (TurA and TurB) encoded on the chromosome are expressed predominantly. Previous transcriptome analyses suggested that TurA, TurB, and Pmr cooperatively regulate numerous genes, but the differentially transcribed genes in KT2440Δ*turA*(pCAR1), KT2440Δ*turB*(pCAR1), and KT2440(pCAR1Δ*pmr*) compared with those in KT2440(pCAR1) were somewhat different. Here, we performed RNA sequencing analyses to compare the differentially transcribed genes after the deletion of *turA* or *turB* in KT2440, and *turA*, *turB* or *pmr* in KT2440(pCAR1). Three pCAR1-free strains (KT2440, KT2440Δ*turA*, KT2440Δ*turB*) and four pCAR1-harboring strains [KT2440(pCAR1), KT2440Δ*turA*(pCAR1), KT2440Δ*turB*(pCAR1), KT2440(pCAR1Δ*pmr*)], grown until the log and stationary phases, were used. In KT2440, TurA was the major H-NS family protein regulating a large number and wide range of genes, and both TurA and TurB were suggested to functionally compensate each other, particularly during the stationary phase. In KT2440(pCAR1), the numbers of differentially transcribed genes after the deletion of *turA* or *turB* drastically increased compared to those in KT2440. Notably, more than half of the differentially transcribed genes in KT2440Δ*turA* and KT2440Δ*turB* did not overlap with those in KT2440Δ*turA*(pCAR1) and KT2440Δ*turB*(pCAR1). This dynamic change could be explained by the acquisition of pCAR1 itself and the expression of Pmr. After pCAR1 was transferred into the host, TurA and TurB could be detached from the chromosome of KT2440 and they could newly bind to pCAR1. Moreover, Pmr could reconstitute the chromosome-binding heteromeric oligomers which were formed by TurA and TurB. Our study revealed that horizontal transfer of a plasmid changes the transcriptional network of the chromosomally encoded H-NS family proteins.

## Introduction

Bacteria have defense systems to protect themselves from foreign-derived, also referred to as xenogeneic, DNA. Transcription-silencing proteins play an important role in the avoidance of fitness cost caused by the immediate expression of such xenogeneic genes. H-NS family proteins, a major family of bacterial nucleoid-associated proteins, are well-known xenogeneic silencers which affect both nucleoid compaction and global gene regulation (Dorman, 2009; Ali et al., 2012; Singh et al., 2016). They preferably bind to AT-rich regions, which are usually acquired by horizontal gene transfer, and repress transcription from those regions (Grainger et al., 2006; Lucchini et al., 2006; Navarre et al., 2006; Oshima et al., 2006). As a result, they promote the integration of xenogeneic genes into host cells with a minimal decrease in competitive fitness (Dorman, 2014). Three major groups of proteins have been well studied in this family: H-NS homologs of enterobacteria, Lsr2 homologs of mycobacteria, and MvaT homologs of pseudomonads (Singh et al., 2016). Lsr2 and MvaT homologs are considered to be functional analogs of H-NS which can complement *hns*-related phenotypes of *Escherichia coli*, though they have low amino acid sequence identity with H-NS (Tendeng et al., 2003; Gordon et al., 2008).

Genes encoding H-NS family proteins are sometimes found on plasmids (Shintani et al., 2015). These proteins are considered to be key factors for the cross-talks between plasmids and the host chromosomes (Nojiri, 2013; Vial and Hommais, 2019). H-NS_*R*__27_ and Sfh are H-NS homologs encoded on the incompatibility (Inc) HI1 plasmid R27 and its derivative pSfR27, respectively (Doyle et al., 2007; Baños et al., 2009). Sfh was the first characterized H-NS homolog encoded on a plasmid; Doyle et al. (2007) found that it has a “stealth” function which allows the plasmid pSfR27 to be transmitted into the host cell with minimal effects on the competitive fitness. Further research clarified that the DNA binding regions of Sfh overlapped completely those of chromosomally encoded H-NS, suggesting that Sfh is a molecular backup of the chromosomally encoded H-NS proteins, which can be detached from their chromosomal binding locations due to the acquisition of the large AT-rich plasmid pSfR27 (Dillon et al., 2010). On the other hand, Baños et al. (2009) showed that H-NS_*R*__27_, which is 98% identical to Sfh in amino acid sequence, is not just a backup of the chromosomally encoded H-NS; they showed that H-NS_*R*__27_ selectively targets horizontally acquired DNA and not core genomic DNA, whereas chromosomally encoded H-NS targets both. H-NS homologs are also found on IncA/C plasmids. Acr2, which is encoded on the multidrug resistance plasmid pAR060302, was shown to bind to AT-rich regions and repress conjugative transfer of a pAR060302 derivative (Lang and Johnson, 2016).

Pmr is an MvaT homolog encoded on the catabolic plasmid pCAR1, which belongs to IncP-7 group (Nojiri, 2012). From transcriptome analyses using *Pseudomonas putida* KT2440 as a host of pCAR1 and pCAR1Δ*pmr*, we previously found that Pmr has a “stealth” function resembling that of Sfh, while transcription levels of some genes on the chromosome and pCAR1 seemed to be specifically regulated by Pmr (Yun et al., 2010). Among the five genes encoding MvaT homologs in KT2440, *turA* and *turB* are considered to be transcribed predominantly, and their protein expression level was previously determined (Yuste et al., 2006; Renzi et al., 2010; Yun et al., 2010; Sun et al., 2017). TurA and TurB are homologs of MvaT and MvaU of *Pseudomonas aeruginosa* PAO1, which bind to almost the same DNA regions and function coordinately (Castang et al., 2008). In our previous study, transcriptome analyses using single-gene deletion mutants of *turA*, *turB*, and *pmr* in KT2440(pCAR1) were performed using custom tiling arrays (Yun et al., 2016). The results revealed that differentially transcribed genes after the deletion of *turA*, *turB*, or *pmr* compared with the wild-type KT2440(pCAR1) were not so overlapped each other, especially when *pmr* was deleted. We also performed chromatin affinity purification coupled with a high-density tiling chip (ChAP-chip) analyses using KT2440(pCAR1) in the same report (Yun et al., 2016). Unexpected from the transcriptome results, the detected binding regions of TurA, TurB, and Pmr were almost the same, probably because the three proteins formed heteromeric oligomers on DNA. Based on the crystal structure of the N-terminal domain of TurB, MvaT homologs have been shown to use two dimerization sites to form higher-order oligomers (Suzuki-Minakuchi et al., 2016).

Here, we prepared single-deletion mutants of *turA* and *turB* in KT2440 to compare transcriptome before and after transfer of pCAR1 into the host. We performed RNA sequencing (RNA-Seq) analyses using three pCAR1-free strains (KT2440, KT2440Δ*turA*, KT2440Δ*turB*) and four pCAR1-harbouring strains [KT2440(pCAR1), KT2440Δ*turA*(pCAR1), KT2440Δ*turB*(pCAR1), KT2440(pCAR1Δ*pmr*)]. We also aimed to clarify how participation of Pmr accompanied by pCAR1 carriage alters the transcriptional regulatory network constructed by TurA and TurB.

## Materials and Methods

### Bacterial Strains, Plasmids, and Culture Conditions

The bacterial strains and plasmids used in this study are listed in Table 1. Markerless deletion mutants of *turA* or *turB* were prepared from KT2440 using pK19mobsacBΔ*turA* or pK19mobsacBΔ*turB* according to the methods described previously (Suzuki-Minakuchi et al., 2015). *E. coli* S17-1λ*pir* was grown at 37°C in lysogeny broth (LB) (g/l: tryptone, 10; yeast extract, 5; NaCl, 10) (Sambrook and Russell, 2001), whereas KT2440 and its derivative strains were grown at 30°C in LB or nitrogen plus mineral medium 4 (NMM-4) (g/l: Na_2_HPO_4_, 2.2; KH_2_PO_4_, 0.8; NH_4_NO_3_, 3.0; MgSO_4_⋅7H_2_O, 0.2; FeCl_3_⋅6H_2_O, 0.01; CaCl_2_⋅2H_2_O, 0.01) (Shintani et al., 2005) containing 0.1% (w/v) succinate as the sole source of carbon and energy. For plate cultures, the medium was solidified using 1.6% (w/v) Agar Purified, powder (Nacalai Tesque, Kyoto, Japan).

**TABLE 1:** Bacterial strains and plasmids used in this study.

**Bacterial strain or plasmid**	**Relevant characteristic(s)**	**Source or reference(s)**
**Bacterial strains**		
*E. coli*		
S17-1λ*pir*	RK2 *tra* regulon, host for *pir*-dependent plasmids, *recA*, *thi*, *pro*, Δ*hsdR*, M^+^, RP4-2-Tc:Mu-Km:Tn*7*, λ*pir*, Tp^r^, Sm^r^	Simon et al. (1983)
*P. putida*		
KT2440	Naturally Cm^r^	Nelson et al. (2002)
KT2440Δ*turA*	KT2440 single-deletion mutant lacking *turA* gene	This study
KT2440Δ*turB*	KT2440 single-deletion mutant lacking *turB* gene	This study
KT2440(pCAR1)	KT2440 harboring pCAR1	Shintani et al. (2006); Miyakoshi et al. (2007)
KT2440Δ*turA*(pCAR1)	KT2440(pCAR1) single-deletion mutant lacking *turA* gene	Yun et al. (2016)
KT2440Δ*turB*(pCAR1)	KT2440(pCAR1) single-deletion mutant lacking *turB* gene	Yun et al. (2016)
KT2440(pCAR1Δ*pmr*)	KT2440(pCAR1) single-deletion mutant lacking *pmr* gene	Suzuki-Minakuchi et al. (2015)
**Plasmids**		
pFLP2Km	Flp recombinase expression vector for removal of Gm^r^ gene, Km^r^, ori1600, oriT(RP4)	Yun et al. (2010)
pK19mobsacBΔ*turA*	pK19mobsacB containing 5′- and 3′-flanking regions of *turA* and Gm^r^ gene, which is flanked by FRT sites	Yun et al. (2016)
pK19mobsacBΔ*turB*	pK19mobsacB containing 5′- and 3′-flanking regions of *turB* and Gm^r^ gene, which is flanked by FRT sites	Yun et al. (2016)

### RNA Extraction

Overnight LB cultures of KT2440 and its derivatives were inoculated into 100 ml of succinate-supplemented NMM-4 to obtain an initial turbidity at 600 nm of 0.025. The cultures were incubated in a rotating shaker at 120 revolutions per minute, and 10 ml and 5 ml of the culture was collected at the log (turbidity at 600 nm of 0.031 to 0.11) and the stationary (turbidity at 600 nm of 0.13 to 0.22) phases, respectively (Supplementary Figure S1). Biologically duplicated samples were prepared for each strain. The cells were treated with RNAprotect Bacteria Reagent (Qiagen, Hilden, Germany) immediately after sampling. RNA extraction was performed using the RNeasy Mini kit (Qiagen) according to the manufacturer’s instructions. Extracted RNA eluates were treated with RQ1 RNase-free DNase (Promega, Madison, WI, United States) and purified again with a second round of the same RNA extraction procedure.

### RNA-Seq Analyses

rRNA was removed by Ribo-Zero rRNA Removal Kits for Bacteria (Illumina, San Diego, CA, United States). RNA-Seq libraries were prepared using the NEBNext Ultra RNA Library Prep Kit for Illumina (New England Biolabs, Ipswich, MA, United States) following the manufacturer’s instructions. Paired-end sequencing (2 × 100 bp) of prepared libraries was performed on a HiSeq2500 sequencer (Illumina). Read trimming and mapping were performed using CLC Genomics Workbench (ver. 7.5.2, CLC Bio, Aarhus, Denmark). Trimming parameters were as follows: quality score limit, 0.001; maximum number of ambiguous nucleotides, 0; number of removed 5′ terminal nucleotides, 15; number of removed 3′ terminal nucleotides, 3; and discard reads below length, 20. Then, the trimmed reads were mapped to the sequences of the *P. putida* KT2440 chromosome (NC_002947.4) and the pCAR1 plasmid (NC_004444.1) with the following parameters: mismatch cost, 2; insertion cost, 3; deletion cost, 3; length fraction, 0.7; similarity fraction, 0.85; auto-detected paired distances, yes; strand specific, both; and maximum number of hits for a read, 1. High correlations of the resultant read count data normalized by reads per kilobase of transcript per million mapped reads (RPKM) between each pair of biological replicates were observed (Supplementary Figure S2).

To detect differentially transcribed genes between two different strains, we used R (ver. 3.5.3)^1^ and EdgeR (Robinson et al., 2010), a Bioconductor package for the analysis of digital gene expression data (ver. 3.24.3)^2^. In EdgeR, replicated read count data were analyzed based on a negative binomial model including normalization factors and dispersion values. The read count data were normalized by the trimmed mean of M-values (TMM) normalization method. Then, common dispersion and moderated tagwise dispersion were calculated by the quantile-adjusted conditional maximum likelihood (qCML) method. By using exactTest in EdgeR, differentially transcribed genes were detected using a false discovery rate (FDR) threshold of less than 0.05 to adjusted *P* values, which were generated using the Benjamini and Hochberg method. Calculation of log_2_-fold-change values was done to distinguish up-regulated genes [log_2_-fold-change > 0] and down-regulated genes [log_2_-fold-change < 0] among the differentially transcribed genes. All the FDR and log_2_-fold-change values calculated for each gene on the KT2440 chromosome are listed in Supplementary Table S1 (log phase) and Supplementary Table S2 (stationary phase).

## Results and Discussion

### Transcriptomic Profiles of KT2440Δ*turA* and KT2440Δ*turB*

**FIGURE 1:**
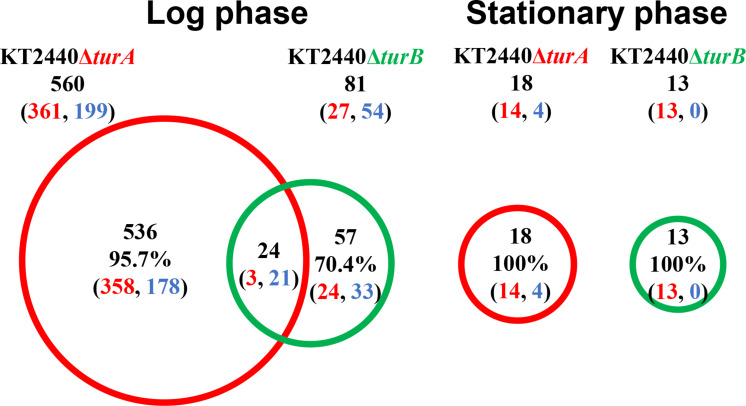
Venn diagram of the differentially transcribed genes in KT2440Δ*turA* and KT2440Δ*turB*. The numbers of differentially transcribed genes in KT2440Δ*turA* and KT2440Δ*turB* compared with the wild-type KT2440 are shown. The numbers of up- and down-regulated genes are shown in red and blue digits, respectively.

First, the transcriptomic profiles of KT2440Δ*turA* and KT2440Δ*turB* were compared to the transcriptomic profile of KT2440. As shown in Figure 1, 560 genes were differentially transcribed in KT2440Δ*turA* in the log phase, while 81 genes were differentially transcribed in KT2440Δ*turB*. This result suggests that TurA regulates a wider range of genes than TurB during the log phase. Similar results were obtained by Renzi et al. (2010) previously. This difference in the numbers of the differentially transcribed genes after the deletion of *turA* or *turB* likely stems from differing numbers of TurA and TurB molecules in the cell. The number of TurA molecules per cell in KT2440 during the log phase was approximately 60,000 molecules, in contrast to the number of TurB molecules per cell, which was less than 1,000 molecules (Sun et al., 2017). On the other hand, during the stationary phase, only 18 and 13 genes were differentially transcribed in KT2440Δ*turA* and KT2440Δ*turB*, respectively (Figure 1). There are two possible explanations for this result. First, TurA and TurB may have difficulty binding to DNA during the stationary phase. Lioy et al. (2018) recently observed that a large-scale chromosomal reorganization takes place in *E. coli* during the transition period between the log and stationary phases. The structural difference in the nucleoid could affect the binding manner of TurA and TurB. Second, TurA and TurB may compensate for each other functionally during the stationary phase. The number of TurB molecules during the stationary phase was approximately 3,000 molecules per cell, and the number of TurA molecules decreased to 25,000 molecules per cell (Sun et al., 2017). Since the difference in the number of TurA and TurB molecules was smaller during the stationary phase than in the log phase, TurA and TurB may complement each other during the stationary phase if one of them is lost from the cell, resulting in a small number of differentially transcribed genes in KT2440Δ*turA* and KT2440Δ*turB*. Moreover, our recent study revealed that the DNA-binding affinity of TurB is higher than that of TurA (data not shown). This is another reason why TurB may compensate for the function of TurA, despite the fact that the number of TurA is larger than that of TurB even during the stationary phase.

**FIGURE 2:**
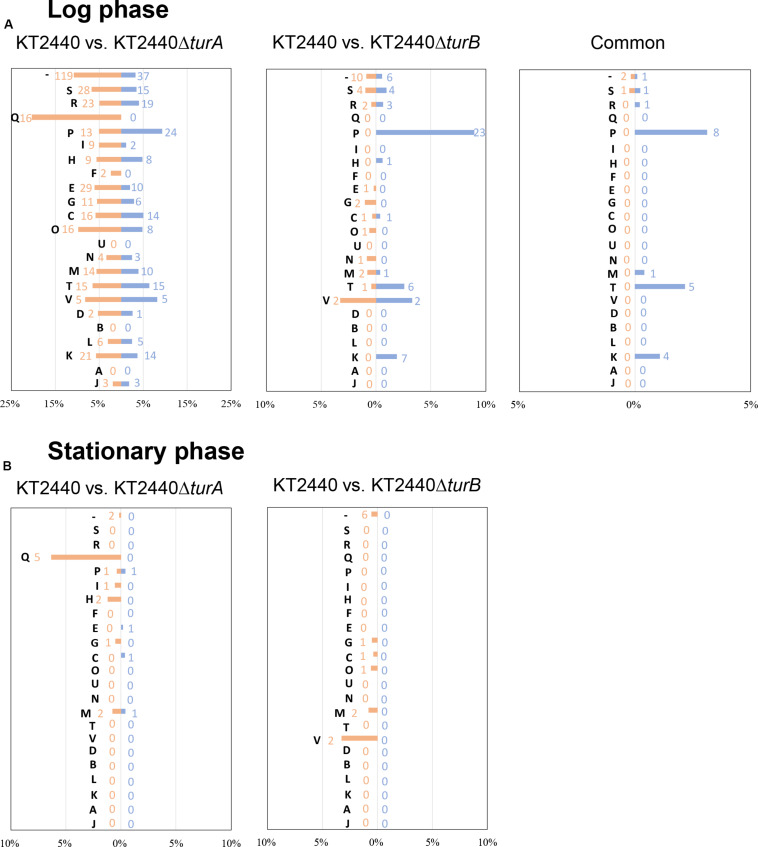
COG analysis of the differentially transcribed genes in KT2440Δ*turA* and KT2440Δ*turB*. The differentially transcribed genes in KT2440Δ*turA*, KT2440Δ*turB*, and both of them in the log phase **(A)** and the stationary phase **(B)** were categorized by COG. Orange bars show the percentages of up-regulated genes by the deletion of *turA* or *turB* which were classified into respective COG categories among all the genes classified into the same COG category. Blue bars show those of the down-regulated genes. The numbers of differentially transcribed genes classified into respective COG categories are also shown in orange and blue. The descriptions for each COG category are as follows: [J]: translation, ribosomal structure and biogenesis, [A]: RNA processing and modification, [K]: transcription, [L]: replication, recombination and repair, [B]: chromatin structure and dynamics, [D]: cell cycle control, cell division, chromosome partitioning, [V]: defense mechanisms, [T]: signal transduction mechanisms, [M]: cell wall/membrane/envelope biogenesis, [N]: cell motility, [U]: intracellular trafficking, secretion, and vesicular transport, [O]: posttranslational modification, protein turnover, chaperones, [C]: energy production and conversion, [G]: carbohydrate transport and metabolism, [E]: amino acid transport and metabolism, [F]: nucleotide transport and metabolism, [H]: coenzyme transport and metabolism, [I]: lipid transport and metabolism, [P]: inorganic ion transport and metabolism, [Q]: secondary metabolites biosynthesis, transport and catabolism, [R]: general function prediction only, [S]: function unknown, [–]: uncharacterized.

Next, to investigate the types of genes that are differentially transcribed in KT2440Δ*turA* and KT2440Δ*turB*, the genes were categorized into Clusters of Orthologous Groups of proteins (COG)^3^ (Figure 2). During the log phase (Figure 2A), TurA affected the transcription of various types of genes, including those classified into the COG categories P (inorganic ion transport and metabolism), E (amino acid transport and metabolism), C (energy production and conversion), O (posttranslational modification, protein turnover, chaperones), M (cell wall/membrane/envelope biogenesis), T (signal transduction mechanisms), and K (transcription). Based on the ratio of COG-categorized, differentially transcribed genes resulting from *turA* deletion to all genes grouped into the same COG categories, TurA was also involved in the regulation of genes in the COG categories Q (secondary metabolites biosynthesis, transport and catabolism) and V (defense mechanisms), though the number of genes included in these categories were not so large. In contrast, TurB was mainly involved in the expression of genes classified into the COG categories P (inorganic ion transport and metabolism), T (signal transduction mechanisms), V (defense mechanisms), and K (transcription). Most of the 24 genes commonly regulated by TurA and TurB (Figure 1) are in the COG categories P (inorganic ion transport and metabolism), T (signal transduction mechanisms), and K (transcription) (Figure 2A). Six of the eight genes in the COG category P (PP_0351, PP_0700, PP_0703, PP_0861, PP_3576, and PP_4612) are predicted to encode FecR homologs and TonB-dependent siderophore receptors (Table 2). Both of them are involved in the transport of ferric citrate (Braun, 1997; Visca et al., 2002). The other genes, assigned to the COG categories T and K, are predicted to encode proteins related to two-component regulatory systems and RNA polymerase sigma factors, respectively (Table 2). These results suggest that TurA and TurB cooperatively regulate the iron-uptake systems and transcriptional networks of the cell.

**TABLE 2:** The 24 genes commonly up- or down-regulated by the deletion of *turA* or *turB* in KT2440 during the log phase.

**Locus tag**	**Gene name**	**COG**	**Annotation**	**Type of regulation**
PP_0270	PP_0270	T	Integral membrane sensor signal transduction histidine kinase	Down-regulated
PP_0271	*gltR-1*	T	Winged helix family two component transcriptional regulator	Down-regulated
PP_0351	PP_0351	P	FecR anti-FecI sigma factor	Down-regulated
PP_0352	PP_0352	K	RNA polymerase sigma factor	Down-regulated
PP_0534	PP_0534	T	Winged helix family two component transcriptional regulator	Down-regulated
PP_0700	PP_0700	P	FecR anti-FecI sigma factor	Down-regulated
PP_0703	PP_0703	P	FecR anti-FecI sigma factor	Down-regulated
PP_0704	PP_0704	K	ECF subfamily RNA polymerase sigma factor	Down-regulated
PP_0861	PP_0861	P	TonB-dependent siderophore receptor	Down-regulated
PP_0862	PP_0862	S	Hydroxylase	Down-regulated
PP_0863	PP_0863	R	Sel1 domain-containing protein repeat-containing protein	Down-regulated
PP_1577	PP_1577	S	Lambda family phage tail tape measure protein	Up-regulated
PP_1651	PP_1651	T	Winged helix family two component transcriptional regulator	Down-regulated
PP_1652	PP_1652	T	Sensor histidine kinase	Down-regulated
PP_2828	PP_2828	–	Hypothetical protein	Up-regulated
PP_3085	PP_3085	P	transmembrane sensor	Down-regulated
PP_3576	PP_3576	P	FecR anti-FecI sigma factor	Down-regulated
PP_3577	PP_3577	K	ECF subfamily RNA polymerase sigma factor	Down-regulated
PP_4609	PP_4609	–	Hypothetical protein	Down-regulated
PP_4611	PP_4611	K	RNA polymerase sigma factor	Down-regulated
PP_4612	PP_4612	P	FecR protein	Down-regulated
PP_5008	PP_5008	–	Poly(hydroxyalkanoate) granule-associated protein	Up-regulated
PP_5139	*cadA-2*	P	Heavy metal translocating P-type ATPase	Down-regulated
PP_5308	*tonB*	M	TonB family protein	Down-regulated

In the stationary phase, five of the 18 genes differentially transcribed in KT2440Δ*turA* belong to the COG category Q (secondary metabolites biosynthesis, transport and catabolism) (Figure 2B). Moreover, 11 of the 14 genes up-regulated during the stationary phase in KT2440Δ*turA* (PP_3161-3168 and PP_3713-3715), including the above five genes, are involved in the degradation of benzoate via the catechol branch of the β-ketoadipate pathway (Harwood and Parales, 1996) (Table 3). In contrast, in KT2440Δ*turB*, a greater proportion of the differentially transcribed genes fell into the COG categories M (cell wall/membrane/envelope biogenesis) and V (defense mechanisms) (Figure 2B). Three of these four genes (PP_3425-3427) constitute the *mexEF*-*oprN* operon (Table 4), which encodes a multidrug efflux pump that is up-regulated by the deletion of *mvaT* in *Pseudomonas aeruginosa* PAO1 (Westfall et al., 2006).

**TABLE 3:** The 18 genes differentially transcribed after the deletion of *turA* in KT2440 during the stationary phase.

**Locus tag**	**Gene name**	**COG**	**Annotation**	**Type of regulation**
PP_0241	*ssuF*	H	TOBE domain-containing protein	Up-regulated
PP_1924	PP_1924	M	Phosphinothricin *N*-acetyltransferase	Up-regulated
PP_3161	benA*	P	Benzoate dioxygenase subunit alpha	Up-regulated
PP_3162	benB*	Q	Benzoate dioxygenase subunit beta	Up-regulated
PP_3163	benC*	H	Oxidoreductase FAD/NAD(P)-binding domain-containing protein	Up-regulated
PP_3164	benD*	I	1,6-Dihydroxycyclohexa-2,4-diene-1-carboxylate dehydrogenase	Up-regulated
PP_3165	benK*	G	Major facilitator superfamily transporter	Up-regulated
PP_3166	PP_3166*	Q	Catechol 1,2-dioxygenase	Up-regulated
PP_3167	benE-2*	Q	Benzoate transporter	Up-regulated
PP_3168	*benF*	–	Benzoate-specific porin	Up-regulated
PP_3713	catA*	Q	Catechol 1,2-dioxygenase	Up-regulated
PP_3714	catC*	Q	Muconolactone isomerase	Up-regulated
PP_3715	catB*	M	Muconate and chloromuconate cycloisomerase	Up-regulated
PP_3782	PP_3782*	–	Hypothetical protein	Up-regulated
PP_4403	*bkdB*	C	Branched-chain alpha-keto acid dehydrogenase subunit E2	Down-regulated
PP_5033	*hutU*	E	Urocanate hydratase	Down-regulated
PP_5139	*cadA-2*	P	Heavy metal translocating P-type ATPase	Down-regulated
PP_5269	*dadX*	M	Alanine racemase	Down-regulated

*These genes were also differentially transcribed after the deletion of *turA* in KT2440(pCAR1) during the stationary phase.

**TABLE 4:** The 13 genes differentially transcribed after the deletion of *turB* in KT2440 during the stationary phase.

**Locus tag**	**Gene name**	**COG**	**Annotation**	**Type of regulation**
PP_1895	PP_1895	V	ABC transporter ATP-binding protein	Up-regulated
PP_2022	PP_2022	–	Hypothetical protein	Up-regulated
PP_2023	PP_2023	O	Glutathione S-transferase	Up-regulated
PP_2646	PP_2646	–	Hypothetical protein	Up-regulated
PP_2647	PP_2647	G	Major facilitator family transporter	Up-regulated
PP_2827	PP_2827	C	Zinc-containing alcohol dehydrogenase	Up-regulated
PP_2828	PP_2828	–	Hypothetical protein	Up-regulated
PP_3425	*mexE*	M	RND family efflux transporter MFP subunit	Up-regulated
PP_3426	*mexF*	V	Hydrophobe/amphiphile efflux-1 (HAE1) family transporter	Up-regulated
PP_3427	*oprN*	M	NodT family RND efflux system outer membrane lipoprotein	Up-regulated
PP_3519	PP_3519	–	Lipoprotein	Up-regulated
PP_3770	PP_3770	–	Hypothetical protein	Up-regulated
PP_4858	PP_4858	–	Hypothetical protein	Up-regulated

### Transcriptomic Profiles of KT2440Δ*turA*(pCAR1) and KT2440Δ*turB*(pCAR1)

Next, the transcriptomic profiles of KT2440Δ*turA*(pCAR1) and KT2440Δ*turB*(pCAR1) were compared to the transcriptomic profile of KT2440(pCAR1). Similar analyses have been performed using tiling arrays (Yun et al., 2016). However, in this study, RNA-Seq analyses were performed to compare the differentially transcribed genes after the deletion of *turA* or *turB* in KT2440(pCAR1) and in KT2440, minimizing the effects of using different methodological approaches. Actually, the differentially transcribed genes in KT2440Δ*turA*(pCAR1) and KT2440Δ*turB*(pCAR1) detected in this study differed from those identified by Yun et al. (2016) (Supplementary Figure S3).

**FIGURE 3:**
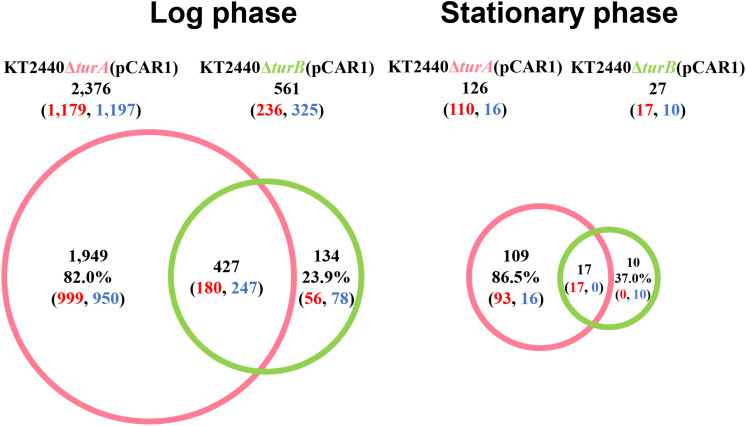
Venn diagram of the differentially transcribed genes in KT2440Δ*turA*(pCAR1) and KT2440Δ*turB*(pCAR1). The numbers of differentially transcribed genes on the chromosome of KT2440Δ*turA*(pCAR1) and KT2440Δ*turB*(pCAR1) compared with the wild-type KT2440(pCAR1) are shown. The numbers of up- and down-regulated genes are shown in red and blue digits, respectively.

In total, 2,376 genes in the log phase and 126 genes in the stationary phase were differentially transcribed in KT2440Δ*turA*(pCAR1), whereas 561 genes in the log phase and 27 genes in the stationary phase were differentially transcribed in KT2440Δ*turB*(pCAR1) (Figure 3). As observed in the plasmid-free KT2440, TurA was involved in the regulation of a wider range of genes than TurB in KT2440(pCAR1), particularly in the log phase. Moreover, the number of differentially transcribed genes was lower in the stationary phase than in the log phase, and a similar tendency was observed in KT2440. Comparing the number of differentially transcribed genes after the deletion of *turA* or *turB* in KT2440 and KT2440(pCAR1), the number was higher in the presence of pCAR1 regardless of the growth phase (Figures 1, 3). This may be because a portion of the population of TurA and TurB proteins, which bound to the KT2440 chromosome, were detached by pCAR1 carriage and they newly bound to pCAR1 instead of the KT2440(pCAR1) chromosome. As a result, the relative amount of TurA and TurB capable of binding the chromosome becomes smaller in KT2440(pCAR1) than in KT2440, and TurA and TurB compensated less well for each other when their counterpart was lost in KT2440(pCAR1), compared to KT2440. This may have led to the increased number of differentially transcribed genes after the deletion of *turA* or *turB* in response to pCAR1 carriage. In addition, the genes that were differentially transcribed in KT2440Δ*turA*(pCAR1) and KT2440Δ*turB*(pCAR1) overlapped more than those in KT2440Δ*turA* and KT2440Δ*turB*, suggesting that the contribution of TurB to cooperative gene regulation is higher with TurA in KT2440(pCAR1) than with TurA in KT2440.

**FIGURE 4:**
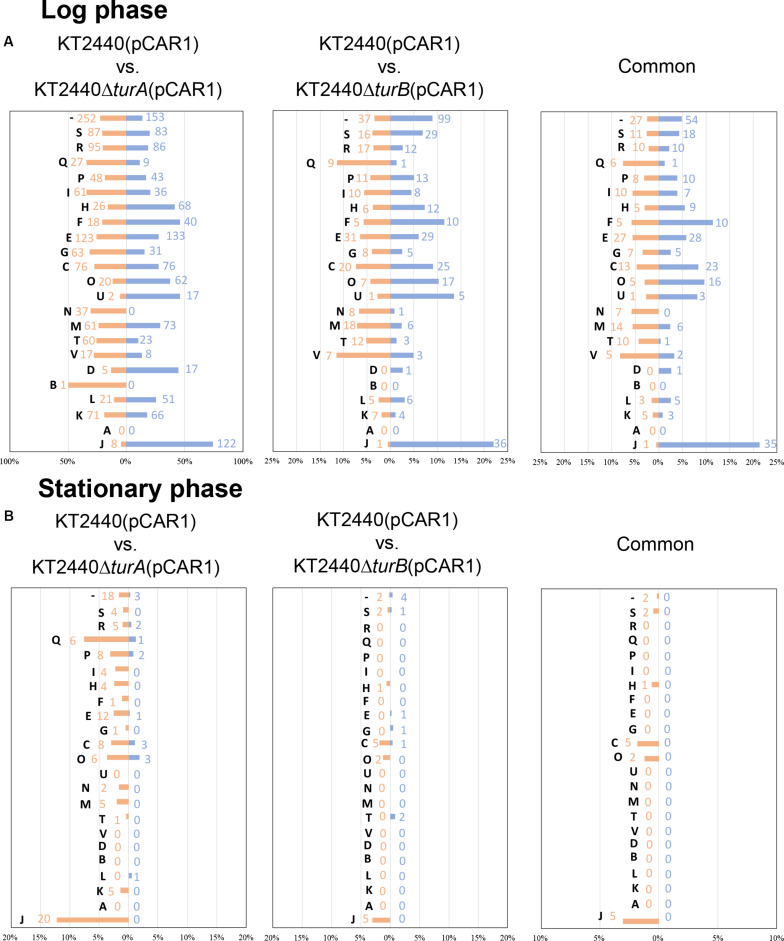
COG analysis of the differentially transcribed genes in KT2440Δ*turA*(pCAR1) and KT2440Δ*turB*(pCAR1). The differentially transcribed genes on the chromosome of KT2440Δ*turA*(pCAR1), KT2440Δ*turB*(pCAR1), and both of them in the log phase **(A)** and the stationary phase **(B)** were categorized by COG. Orange bars show the percentages of up-regulated genes by the deletion of *turA* or *turB* which were classified into respective COG categories among all the genes classified into the same COG category. Blue bars show those of the down-regulated genes. The numbers of the differentially transcribed genes which were classified into respective COG categories are also shown in orange and blue. The descriptions for each COG category are shown in Figure 2.

Next, the genes that were differentially transcribed in KT2440Δ*turA*(pCAR1) and KT2440Δ*turB*(pCAR1) in the log phase were categorized into the COG groups (Figure 4A) and compared to the results from KT2440 (Figure 2A). Like the KT2440 results, the differentially transcribed genes in KT2440Δ*turA*(pCAR1) were distributed across a wide range of COG categories. Many were grouped into COG categories that were not significantly represented in KT2440, including the following categories: I (lipid transport and metabolism), H (coenzyme transport and metabolism), F (nucleotide transport and metabolism), G (carbohydrate transport and metabolism), U (intracellular trafficking, secretion, and vesicular transport), N (cell motility), D (cell cycle control, cell division, chromosome partitioning), B (chromatin structure and dynamics), L (replication, recombination and repair), and J (translation, ribosomal structure and biogenesis). The differences were more significant when the genes that were differentially transcribed in KT2440Δ*turB*(pCAR1) and those in KT2440Δ*turB* were compared. The differentially transcribed genes in KT2440Δ*turB*(pCAR1) were distributed in most of the COG categories, except for categories A (RNA processing and modification) and B (chromatin structure and dynamics) (Figure 4A). This finding suggests that TurA and TurB functionally complement each other in KT2440(pCAR1), as discussed above. Moreover, a high percentage of the genes classified into category J (translation, ribosomal structure and biogenesis) were down-regulated by the deletion of *turA* or *turB* in KT2440(pCAR1). In KT2440, only three of the 164 genes assigned to category J were down-regulated by the deletion of *turA*, whereas none were affected by the deletion of *turB* (Figure 2A). In contrast, 122 and 36 of the 164 genes grouped in category J were down-regulated in KT2440(pCAR1) due to the deletion of *turA* and *turB*, respectively (Figure 4A). Among the genes commonly regulated by TurA and TurB, 35 genes categorized into the COG category J were down-regulated in KT2440(pCAR1) (Figure 4A). Finally, almost all of the 35 genes were predicted to encode ribosomal proteins, or proteins related to tRNA function (Table 5). These results suggest that TurA and TurB cooperatively enhance the expression of translation-related proteins. Since pCAR1 has approximately 200 genes, it is possible that the number of ribosomes and other translation-related proteins relative to the total number of genes is insufficient when pCAR1 is transferred into KT2440. Thus, the role of TurA and TurB in enhancing the expression levels of translation-related proteins could be helpful in maintaining the expression levels of the chromosomally encoded proteins, thereby counteracting the physiological changes resulting from pCAR1 carriage.

**TABLE 5:** The 35 genes commonly down-regulated by the deletion of *turA* or *turB* in KT2440(pCAR1) categorized in COG category J during the log phase.

**Locus tag**	**Gene name**	**Annotation**
PP_0008	*rnpA*	Ribonuclease P
PP_0389	*rpsU*	30S ribosomal protein S21
PP_0443	*rplK*	50S ribosomal protein L11
PP_0446	*rplL*	50S ribosomal protein L7/L12
PP_0449	*rpsL*	30S ribosomal protein S12
PP_0453	*rpsJ*	30S ribosomal protein S10
PP_0458	*rpsS*	30S ribosomal protein S19
PP_0459	*rplV*	50S ribosomal protein L22
PP_0460	*rpsC*	30S ribosomal protein S3
PP_0463	*rpsQ*	30S ribosomal protein S17
PP_0469	*rplF*	50S ribosomal protein L6
PP_0472	*rpmD*	50S ribosomal protein L30
PP_0473	*rplO*	50S ribosomal protein L15
PP_0475	*rpmJ*	50S ribosomal protein L36
PP_0476	*rpsM*	30S ribosomal protein S13
PP_0477	*rpsK*	30S ribosomal protein S11
PP_0478	*rpsD*	30S ribosomal protein S4
PP_0600	*rpsT*	30S ribosomal protein S20
PP_0689	*rpmA*	50S ribosomal protein L27
PP_1462	*rpsP*	30S ribosomal protein S16
PP_1463	*rim*	16S rRNA-processing protein RimM
PP_1496	*lysS*	Lysyl-tRNA synthetase
PP_1911	*rpmF*	50S ribosomal protein L32
PP_2465	*thrS*	Threonyl-tRNA synthetase
PP_2466	*infC*	Translation initiation factor IF-3
PP_2467	*rpmI*	50S ribosomal protein L35
PP_4007	*infA*	Translation initiation factor IF-1
PP_4794	*leuS*	Leucyl-tRNA synthetase
PP_4818	*prmA*	50S ribosomal protein L11 methyltransferase
PP_4876	*rpsR*	30S ribosomal protein S18
PP_4877	*rpsF*	30S ribosomal protein S6
PP_5027	*dtD*	D-Tyrosyl-tRNA(Tyr) deacylase
PP_5087	*rpmE*	50S ribosomal protein L31
PP_5281	*rpmG*	50S ribosomal protein L33
PP_5282	*rpmB*	50S ribosomal protein L28

Next, we sorted the differentially transcribed genes in KT2440Δ*turA*(pCAR1) and KT2440Δ*turB*(pCAR1) in the stationary phase into the COG categories (Figure 4B). The differentially transcribed genes in KT2440Δ*turA*(pCAR1) come from 18 COG categories, whereas those in KT2440Δ*turB*(pCAR1) are from only nine categories, all of which were included in the 18 COG categories of KT2440Δ*turA*(pCAR1). When genes regulated by both TurA and TurB were categorized by COG, all of the genes up-regulated by the deletion of *turB* in KT2440(pCAR1) were also up-regulated by the deletion of *turA* in KT2440(pCAR1). In contrast, none of the genes that were down-regulated by the deletion of *turB* were down-regulated by the deletion of *turA* in KT2440(pCAR1). These results suggest that TurA and TurB work cooperatively as repressors, and that TurB may play a different role than TurA in inducing gene expression. A high percentage of the genes that were differentially transcribed in KT2440Δ*turA*(pCAR1) and KT2440Δ*turB*(pCAR1) during the stationary phase are from the COG category J (translation, ribosomal structure and biogenesis). Five of the differentially transcribed genes in KT2440Δ*turB*(pCAR1) assigned to this category were also differentially transcribed in KT2440Δ*turA*(pCAR1). In contrast to the results in the log phase, all of the genes grouped in COG category J during the stationary phase were up-regulated. Moreover, most of the differentially transcribed genes in KT2440Δ*turA*(pCAR1) in category J during the stationary phase were genes encoding ribosomal proteins or other translation-related proteins, and were down-regulated during the log phase (Table 6). These results suggest that TurA and TurB repress the expression of translation-related proteins in the stationary phase, when translation activity becomes lower compared to the log phase. The genes that were differentially transcribed after the deletion of *turA* or *turB* in KT2440(pCAR1) during the stationary phase (Figure 4B) were compared to those in KT2440 (Figure 2B). The differentially transcribed genes in KT2440Δ*turA*(pCAR1) were distributed across a wider range of COG categories than those in KT2440Δ*turA*. Among the 18 genes differentially transcribed in KT2440Δ*turA* during the stationary phase, 11 genes were also differentially transcribed in KT2440Δ*turA*(pCAR1) (Table 3). Ten (PP_3161-3167 and PP_3713-3715) are involved in the β-ketoadipate pathway mentioned above, which suggests that TurA is involved in the regulation of these genes regardless of the presence of pCAR1. In contrast, none of the 13 genes differentially transcribed in KT2440Δ*turB* during the stationary phase (Table 4) was differentially transcribed in KT2440Δ*turB*(pCAR1) during this phase.

**TABLE 6:** The 20 genes up-regulated by the deletion of *turA* in KT2440(pCAR1) categorized in COG category J during the stationary phase.

**Locus tag**	**Gene name**	**Annotation**
PP_0449	*rpsL*^a,c^	30S ribosomal protein S12
PP_0456	*rplW*^a^	50S ribosomal protein L23
PP_0459	*rplV*^a^	50S ribosomal protein L22
PP_0463	*rpsQ*^a^	30S ribosomal protein S17
PP_0464	*rplN*^a,c^	50S ribosomal protein L14
PP_0465	*rplX*^a^	50S ribosomal protein L24
PP_0477	*rpsK*^a^	30S ribosomal protein S11
PP_0478	*rpsD*^a^	30S ribosomal protein S4
PP_0688	*rplU*^a^	50S ribosomal protein L21
PP_1464	*trmD*^a^	tRNA (guanine-*N*(1)-)-methyltransferase
PP_1465	*rplS*^a,c^	50S ribosomal protein L19
PP_1858	*efp*^a,c^	Elongation factor P
PP_2467	*rpmI*^a^	50S ribosomal protein L35
PP_3777	PP_3777^b^	Hypothetical protein
PP_3784	PP_3784^b^	Hypothetical protein
PP_4709	*rpsO*^a^	30S ribosomal protein S15
PP_4876	*rpsR*^a^	30S ribosomal protein S18
PP_4877	*rpsF*^a^	30S ribosomal protein S6
PP_5087	*rpmE*^a^	50S ribosomal protein L31
PP_5282	*rpmB*^a,c^	50S ribosomal protein L28

*^*a*^Down-regulated genes by the deletion of *turA* in KT2440(pCAR1) during the log phase. ^*b*^Up-regulated genes by the deletion of *turA* in KT2440(pCAR1) during the log phase. ^*c*^Up-regulated genes by the deletion of *turB* in KT2440(pCAR1) during the stationary phase.*

### Comparison of the Transcriptomic Profiles of pCAR1-Free and pCAR1-Harboring Strains

**FIGURE 5:**
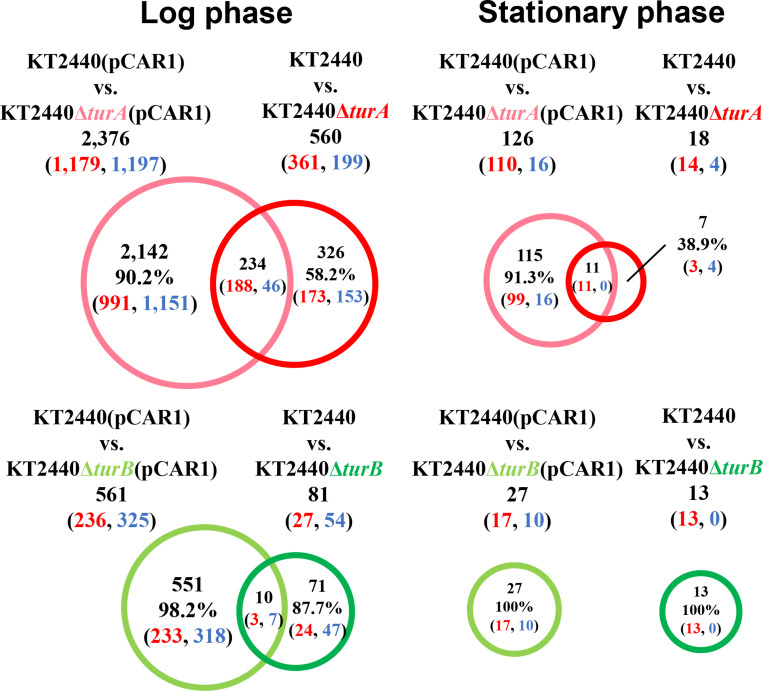
Venn diagram of the differentially transcribed genes after the deletion of *turA* or *turB* in KT2440 and KT2440(pCAR1). The numbers of differentially transcribed genes in KT2440Δ*turA* and KT2440Δ*turB* compared with the wild-type KT2440, and those on the chromosome of KT2440Δ*turA*(pCAR1) and KT2440Δ*turB*(pCAR1) compared with the wild-type KT2440(pCAR1) are shown. The numbers of up- and down-regulated genes are shown in red and blue digits, respectively.

**FIGURE 6:**
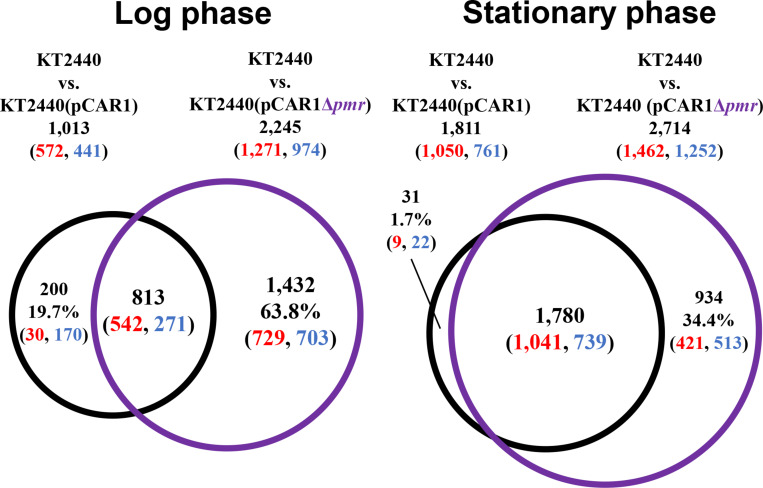
Venn diagram of the differentially transcribed genes due to the transfer of pCAR1 or pCAR1Δ*pmr* into KT2440. The numbers of differentially transcribed genes on the chromosome of KT2440(pCAR1) and KT2440(pCAR1Δ*pmr*) compared with KT2440 are shown. The numbers of up- and down-regulated genes are shown in red and blue digits, respectively.

**FIGURE 7:**
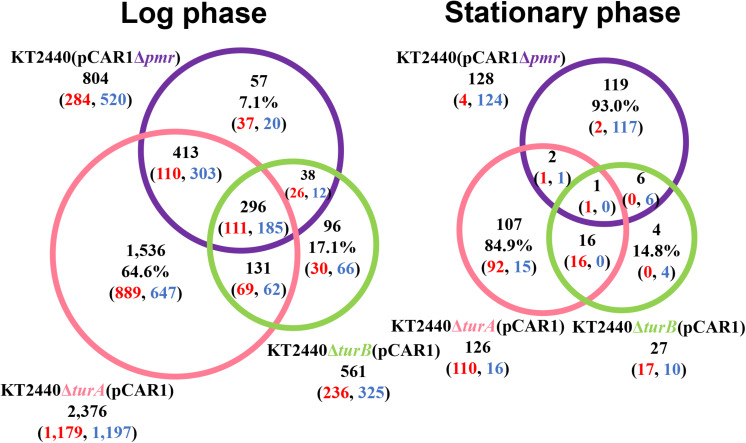
Venn diagram of the differentially transcribed genes in KT2440Δ*turA*(pCAR1), KT2440Δ*turB*(pCAR1), and KT2440(pCAR1Δ*pmr*). The numbers of differentially transcribed genes on the chromosome of KT2440Δ*turA*(pCAR1), KT2440Δ*turB*(pCAR1), and KT2440(pCAR1Δ*pmr*) compared with the wild-type KT2440(pCAR1) are shown. The numbers of up- and down-regulated genes are shown in red and blue digits, respectively.

To better understand the effect of pCAR1 carriage, the overlap in the differentially transcribed genes after the deletion of *turA* or *turB* between KT2440 and KT2440(pCAR1) was studied (Figure 5). Surprisingly, more than half of the genes differentially transcribed after the deletion of *turA* or *turB* in KT2440 did not overlap with those in KT2440(pCAR1). The exception was the genes that were differentially transcribed after the deletion of *turA* in the stationary phase. These results clearly show that the genes differentially transcribed after the deletion of *turA* or *turB* were drastically changed by pCAR1 carriage. We propose two possible explanations for this. First, the drastic change may have been attributable to the detachment of TurA and TurB from the chromosome due to pCAR1 carriage, which we already mentioned above. Since the differentially transcribed genes in KT2440Δ*turA* and KT2440Δ*turB* did not completely overlap those of KT2440Δ*turA*(pCAR1) and KT2440Δ*turB*(pCAR1) (Figure 5), pCAR1 carriage may alter the binding regions of TurA and TurB on the genome. Second, in addition to the effect of pCAR1 carriage itself, Pmr, the additional MvaT homolog expressed from pCAR1, may have contributed to the drastic change in the differentially transcribed genes after the deletion of *turA* or *turB* between KT2440 and KT2440(pCAR1). One of the important functions of Pmr is a “stealth” function that allows pCAR1 to be transferred into KT2440 with minimal effects on the transcriptomic profiles caused by pCAR1 carriage (Yun et al., 2010). The “stealth” function of Pmr was likewise confirmed in the present study, given that the number of differentially transcribed genes in KT2440(pCAR1Δ*pmr*) was much higher than the number in KT2440(pCAR1) (Figure 6). As a result, Pmr may function as a molecular backup to TurA and TurB. Our previous study showed that Pmr numbers approximately 30,000 molecules per cell in both the log and stationary phases, which is almost the same expression level as that of TurA (Sun et al., 2017). Thus, Pmr might work to enlarge the pool of MvaT homologs in the cell. Nevertheless, the ability of Pmr to do so may still be insufficient considering that a higher number and wider range of genes were differentially transcribed after the deletion of *turA* or *turB* in KT2440(pCAR1) (Figures 3, 4). Another previous study also showed that the differentially transcribed genes in KT2440Δ*turA*(pCAR1), KT2440Δ*turB*(pCAR1), and KT2440(pCAR1Δ*pmr*) are somewhat different (Yun et al., 2016). Although similar results were obtained in the present study (Figure 7), the ratio and number of genes that overlapped among the three strains were not identical to those in the previous study. The genes differentially transcribed among KT2440Δ*turA*(pCAR1), KT2440Δ*turB*(pCAR1), and KT2440(pCAR1Δ*pmr*) may be explained by a difference in the affinity of protein-protein interactions. It was recently found that these three proteins have different affinities when they form heterodimers (Sun et al., 2018). These differences may contribute to determining the genes that are regulated by MvaT homologs in KT2440(pCAR1), in which Pmr participates in the formation of heteromeric oligomers with TurA and TurB.

## Conclusion

In this study, we found that the genes differentially transcribed after the deletion of *turA* or *turB* in KT2440(pCAR1) were different from those in KT2440. This difference could be caused by the acquisition of the large pCAR1 plasmid, which increased the number of binding sites for TurA and TurB. As previously reported, regions of more than 40 kb and 20 kb on pCAR1 (200 kb) have been determined to be binding regions for TurA and TurB, respectively (Yun et al., 2016). These numbers correspond to around one-tenth (for TurA) and one-twentieth (for TurB) of the binding regions on the chromosome (Yun et al., 2016), suggesting that pCAR1 carriage causes a portion of the TurA and TurB populations to be detached from their chromosomal binding sites. To examine this possibility, it is necessary to compare the binding regions of TurA and TurB in KT2440 and KT2440(pCAR1). Alternatively, the expression of Pmr from pCAR1 could contribute to the drastic change in the genes that were differentially transcribed after the deletion of *turA* or *turB* in KT2440 and KT2440(pCAR1). The differentially transcribed genes in KT2440(pCAR1Δ*pmr*) were not identical to those in KT2440Δ*turA*(pCAR1) or KT2440Δ*turB*(pCAR1). Considering that Pmr has different affinities in forming heterodimers with TurA and TurB (Sun et al., 2018), Pmr may act to reconstitute the heteromeric oligomers formed by TurA and TurB, resulting in a drastic change in the genes regulated by the three MvaT homologs following the acquisition of pCAR1. However, it is unknown whether the transfer of pCAR1 itself or the expression of Pmr is the major cause of the change in the differentially transcribed genes after the deletion of *turA* or *turB* in KT2440 and KT2440(pCAR1). To determine the cause, further RNA-Seq analyses using double mutant strains such as KT2440Δ*turA*(pCAR1Δ*pmr*) and KT2440Δ*turB*(pCAR1Δ*pmr*) are needed. Such analyses will shed light on a new aspect of plasmid transfer whereby an additional homolog of H-NS family proteins is brought into the host.

## Data Availability Statement

Sequencing data of this study have been deposited in the Sequence Read Archive (https://www.ncbi.nlm.nih.gov/sra/) under project number PRJNA602340.

## Author Contributions

TN and HK prepared the samples. YK and SK performed sequencing. TN and CS-M analyzed the data and drafted the manuscript. KO and HN gave overall supervision and coordination, and revised the manuscript. All authors read and approved the final manuscript.

## Conflict of Interest

The authors declare that the research was conducted in the absence of any commercial or financial relationships that could be construed as a potential conflict of interest.

## Footnotes

1 https://www.r-project.org

2 http://bioconductor.org

3 https://www.ncbi.nlm.nih.gov/COG

## References

[B1] Ali S. S. Xia B. Liu J. Navarre W. W. (2012). Silencing of foreign DNA in bacteria. *Curr. Opin. Microbiol.* 15 175–181. 10.1016/j.mib.2011.12.014 22265250

[B2] Baños R. C. Vivero A. Aznar S. García J. Pons M. Madrid C. (2009). Differential regulation of horizontally acquired and core genome genes by the bacterial modulator H-NS. *PLoS Genet.* 5:e1000513. 10.1371/journal.pgen.1000513 19521501 PMC2686267

[B3] Braun V. (1997). Surface signaling: novel transcription initiation mechanism starting from the cell surface. *Arch. Microbiol.* 167 325–331. 10.1007/s002030050451 9148773

[B4] Castang S. McManus H. R. Turner K. H. Dove S. L. (2008). H-NS family members function coordinately in an opportunistic pathogen. *Proc. Natl. Acad. Sci. U.S.A.* 105 18947–18952. 10.1073/pnas.0808215105 19028873 PMC2596223

[B5] Dillon S. C. Cameron A. D. Hokamp K. Lucchini S. Hinton J. C. Dorman C. J. (2010). Genome-wide analysis of the H-NS and Sfh regulatory networks in *Salmonella* typhimurium identifies a plasmid-encoded transcription silencing mechanism. *Mol. Microbiol.* 76 1250–1265. 10.1111/j.1365-2958.2010.07173.x 20444106

[B6] Dorman C. J. (2009). Regulatory integration of horizontally-transferred genes in bacteria. *Front. Biosci.* 14 4103–4112. 10.2741/3515 19273337

[B7] Dorman C. J. (2014). H-NS-like nucleoid-associated proteins, mobile genetic elements and horizontal gene transfer in bacteria. *Plasmid* 75 1–11. 10.1016/j.plasmid.2014.06.004 24998344

[B8] Doyle M. Fookes M. Ivens A. Mangan M. W. Wain J. Dorman C. J. (2007). An H-NS-like stealth protein aids horizontal DNA transmission in bacteria. *Science* 315 251–252. 10.1126/science.1137550 17218529

[B9] Gordon B. R. Imperial R. Wang L. Navarre W. W. Liu J. (2008). Lsr2 of *Mycobacterium* represents a novel class of H-NS-like proteins. *J. Bacteriol.* 190 7052–7059. 10.1128/JB.00733-08 18776007 PMC2580683

[B10] Grainger D. C. Hurd D. Goldberg M. D. Busby S. J. (2006). Association of nucleoid proteins with coding and non-coding segments of the *Escherichia coli* genome. *Nucleic Acids Res.* 34 4642–4652. 10.1093/nar/gkl542 16963779 PMC1636352

[B11] Harwood C. S. Parales R. E. (1996). The beta-ketoadipate pathway and the biology of self-identity. *Annu. Rev. Microbiol.* 50 553–590. 10.1146/annurev.micro.50.1.553 8905091

[B12] Lang K. S. Johnson T. J. (2016). Characterization of Acr2, an H-NS-like protein encoded on A/C2-type plasmids. *Plasmid* 87-88 17–27. 10.1016/j.plasmid.2016.07.004 27492737

[B13] Lioy V. S. Cournac A. Marbouty M. Duigou S. Mozziconacci J. Espeli O. (2018). Multiscale structuring of the *E. coli* chromosome by nucleoid-associated and condensin proteins 18. *Cell* 172 771.e18–783.e18. 10.1016/j.cell.2017.12.027 29358050

[B14] Lucchini S. Rowley G. Goldberg M. D. Hurd D. Harrison M. Hinton J. C. (2006). H-NS mediates the silencing of laterally acquired genes in bacteria. *PLoS Pathog.* 2:e0020081. 10.1371/journal.ppat.0020081 16933988 PMC1550270

[B15] Miyakoshi M. Shintani M. Terabayashi T. Kai S. Yamane H. Nojiri H. (2007). Transcriptome analysis of *Pseudomonas putida* KT2440 harboring the completely sequenced IncP-7 plasmid pCAR1. *J. Bacteriol.* 189 6849–6860. 10.1128/jb.00684-07 17675379 PMC2045235

[B16] Navarre W. W. Porwollik S. Wang Y. McClelland M. Rosen H. Libby S. J. (2006). Selective silencing of foreign DNA with low GC content by the H-NS protein in *Salmonella*. *Science* 313 236–238. 10.1126/science.1128794 16763111

[B17] Nelson K. E. Weinel C. Paulsen I. T. Dodson R. J. Hilbert H. Martins dos Santos V. A. (2002). Complete genome sequence and comparative analysis of the metabolically versatile *Pseudomonas putida* KT2440. *Environ. Microbiol.* 4 799–808. 10.1046/j.1462-2920.2002.00366.x 12534463

[B18] Nojiri H. (2012). Structural and molecular genetic analyses of the bacterial carbazole degradation system. *Biosci. Biotechnol. Biochem.* 76 1–18. 10.1271/bbb.110620 22232235

[B19] Nojiri H. (2013). Impact of catabolic plasmids on host cell physiology. *Curr. Opin. Biotechnol.* 24 423–430. 10.1016/j.copbio.2012.09.014 23083971

[B20] Oshima T. Ishikawa S. Kurokawa K. Aiba H. Ogasawara N. (2006). *Escherichia coli* histone-like protein H-NS preferentially binds to horizontally acquired DNA in association with RNA polymerase. *DNA Res.* 13 141–153. 10.1093/dnares/dsl009 17046956

[B21] Renzi F. Rescalli E. Galli E. Bertoni G. (2010). Identification of genes regulated by the MvaT-like paralogues TurA and TurB of *Pseudomonas putida* KT2440. *Environ. Microbiol.* 12 254–263. 10.1111/j.1462-2920.2009.02064.x 19788653

[B22] Robinson M. D. McCarthy D. J. Smyth G. K. (2010). edgeR: a Bioconductor package for differential expression analysis of digital gene expression data. *Bioinformatics* 26 139–140. 10.1093/bioinformatics/btp616 19910308 PMC2796818

[B23] Sambrook J. Russell D. (2001). *Molecular Cloning: A Laboratory Manual*, 3rd Edn Cold Spring Harbor: Cold Spring Harbor Laboratory Press.

[B24] Shintani M. Habe H. Tsuda M. Omori T. Yamane H. Nojiri H. (2005). Recipient range of IncP-7 conjugative plasmid pCAR2 from *Pseudomonas putida* HS01 is broader than from other *Pseudomonas* strains. *Biotechnol. Lett.* 27 1847–1853. 10.1007/s10529-005-3892-1 16328978

[B25] Shintani M. Suzuki-Minakuchi C. Nojiri H. (2015). Nucleoid-associated proteins encoded on plasmids: occurrence and mode of function. *Plasmid* 80 32–44. 10.1016/j.plasmid.2015.04.008 25952329

[B26] Shintani M. Yano H. Habe H. Omori T. Yamane H. Tsuda M. (2006). Characterization of the replication, maintenance, and transfer features of the IncP-7 plasmid pCAR1, which carries genes involved in carbazole and dioxin degradation. *Appl. Environ. Microbiol.* 72 3206–3216. 10.1128/aem.72.5.3206-3216.2006 16672459 PMC1472330

[B27] Simon R. Priefer U. Pühler A. (1983). A broad host range mobilization system for *in vivo* genetic engineering: transposon mutagenesis in Gram negative bacteria. *Bio Technol.* 1 784–791. 10.1038/nbt1183-784

[B28] Singh K. Milstein J. N. Navarre W. W. (2016). Xenogeneic silencing and its impact on bacterial genomes. *Annu. Rev. Microbiol.* 70 199–213. 10.1146/annurev-micro-102215-095301 27359215

[B29] Sun Z. Vasileva D. Suzuki-Minakuchi C. Okada K. Luo F. Igarashi Y. (2017). Growth phase-dependent expression profiles of three vital H-NS family proteins encoded on the chromosome of *Pseudomonas putida* KT2440 and on the pCAR1 plasmid. *BMC Microbiol.* 17:188. 10.1186/s12866-017-1091-6 28851281 PMC5576294

[B30] Sun Z. Vasileva D. Suzuki-Minakuchi C. Okada K. Luo F. Igarashi Y. (2018). Differential protein-protein binding affinities of H-NS family proteins encoded on the chromosome of *Pseudomonas putida* KT2440 and IncP-7 plasmid pCAR1. *Biosci. Biotechnol. Biochem.* 82 1640–1646. 10.1080/09168451.2018.1484277 29924693

[B31] Suzuki-Minakuchi C. Hirotani R. Shintani M. Takeda T. Takahashi Y. Matsui K. (2015). Effects of three different nucleoid-associated proteins encoded on IncP-7 plasmid pCAR1 on host *Pseudomonas putida* KT2440. *Appl. Environ. Microbiol.* 81 2869–2880. 10.1128/AEM.00023-15 25681185 PMC4375338

[B32] Suzuki-Minakuchi C. Kawazuma K. Matsuzawa J. Vasileva D. Fujimoto Z. Terada T. (2016). Structural similarities and differences in H-NS family proteins revealed by the N-terminal structure of TurB in *Pseudomonas putida* KT2440. *FEBS Lett.* 590 3583–3594. 10.1002/1873-3468.12425 27709616

[B33] Tendeng C. Soutourina O. A. Danchin A. Bertin P. N. (2003). MvaT proteins in *Pseudomonas* spp.: a novel class of H-NS-like proteins. *Microbiology* 149(Pt 11), 3047–3050. 10.1099/mic.0.C0125-0 14600217

[B34] Vial L. Hommais F. (2019). Plasmid-chromosome cross-talks. *Environ. Microbiol.* 22 540–556. 10.1111/1462-2920.14880 31782608

[B35] Visca P. Leoni L. Wilson M. J. Lamont I. L. (2002). Iron transport and regulation, cell signalling and genomics: lessons from *Escherichia coli* and *Pseudomonas*. *Mol. Microbiol.* 45 1177–1190. 10.1046/j.1365-2958.2002.03088.x 12207687

[B36] Westfall L. W. Carty N. L. Layland N. Kuan P. Colmer-Hamood J. A. Hamood A. N. (2006). mvaT mutation modifies the expression of the *Pseudomonas aeruginosa* multidrug efflux operon mexEF-oprN. *FEMS Microbiol. Lett.* 255 247–254. 10.1111/j.1574-6968.2005.00075.x 16448502

[B37] Yun C. S. Suzuki C. Naito K. Takeda T. Takahashi Y. Sai F. (2010). Pmr, a histone-like protein H1 (H-NS) family protein encoded by the IncP-7 plasmid pCAR1, is a key global regulator that alters host function. *J. Bacteriol.* 192 4720–4731. 10.1128/JB.00591-10 20639326 PMC2937398

[B38] Yun C. S. Takahashi Y. Shintani M. Takeda T. Suzuki-Minakuchi C. Okada K. (2016). MvaT family proteins encoded on IncP-7 plasmid pCAR1 and the host chromosome regulate the host transcriptome cooperatively but differently. *Appl. Environ. Microbiol.* 82 832–842. 10.1128/AEM.03071-15 26590283 PMC4725287

[B39] Yuste L. Hervas A. B. Canosa I. Tobes R. Jimenez J. I. Nogales J. (2006). Growth phase-dependent expression of the *Pseudomonas putida* KT2440 transcriptional machinery analysed with a genome-wide DNA microarray. *Environ. Microbiol.* 8 165–177. 10.1111/j.1462-2920.2005.00890.x 16343331

