# Supplementary material for: H-NS Family Proteins Drastically Change Their Targets in Response to the Horizontal Transfer of the Catabolic Plasmid pCAR1
Source: Front Microbiol. 2020 May 29;11:1099. doi: 10.3389/fmicb.2020.01099 (PMC7273181; doi:10.3389/fmicb.2020.01099)
Supplement: Supplementary file 1 [file Image_1.PDF]

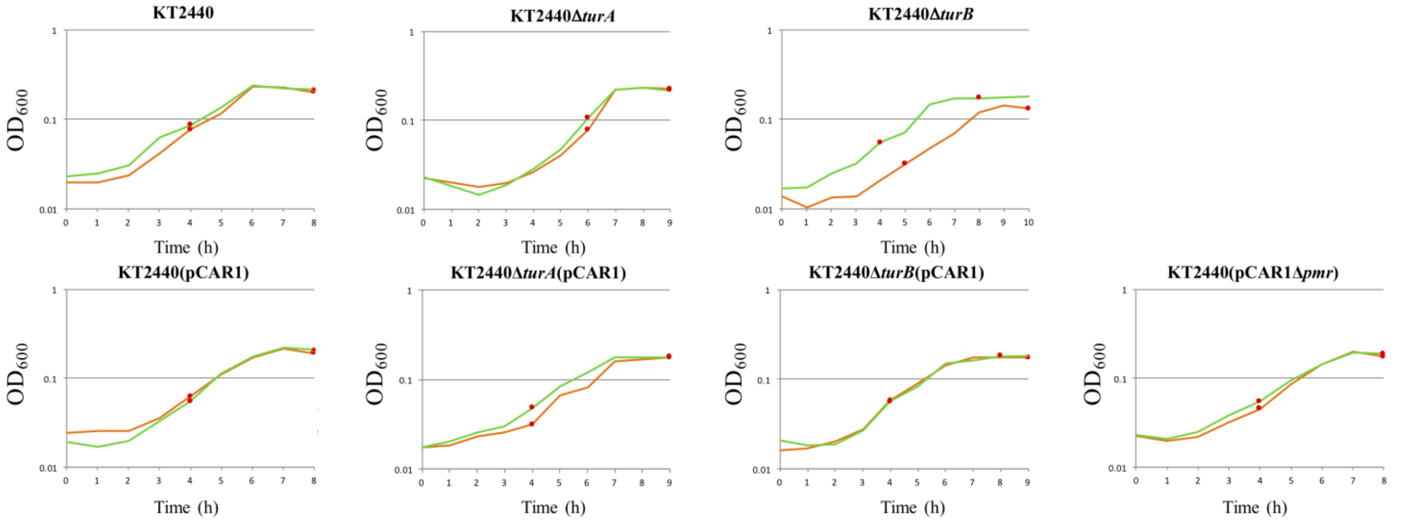

Supplementary Figure S1. Growth curves and sampling points. Growth curves of all the seven strains which were used for RNA extraction are shown. The Y-axis represents OD_{600} of the culture and X-axis represents incubation time (hour). Red dots indicate sampling points. For each strain, orange and light green lines show the growth curves of the first (#1) and second (#2) biological replicates, respectively.

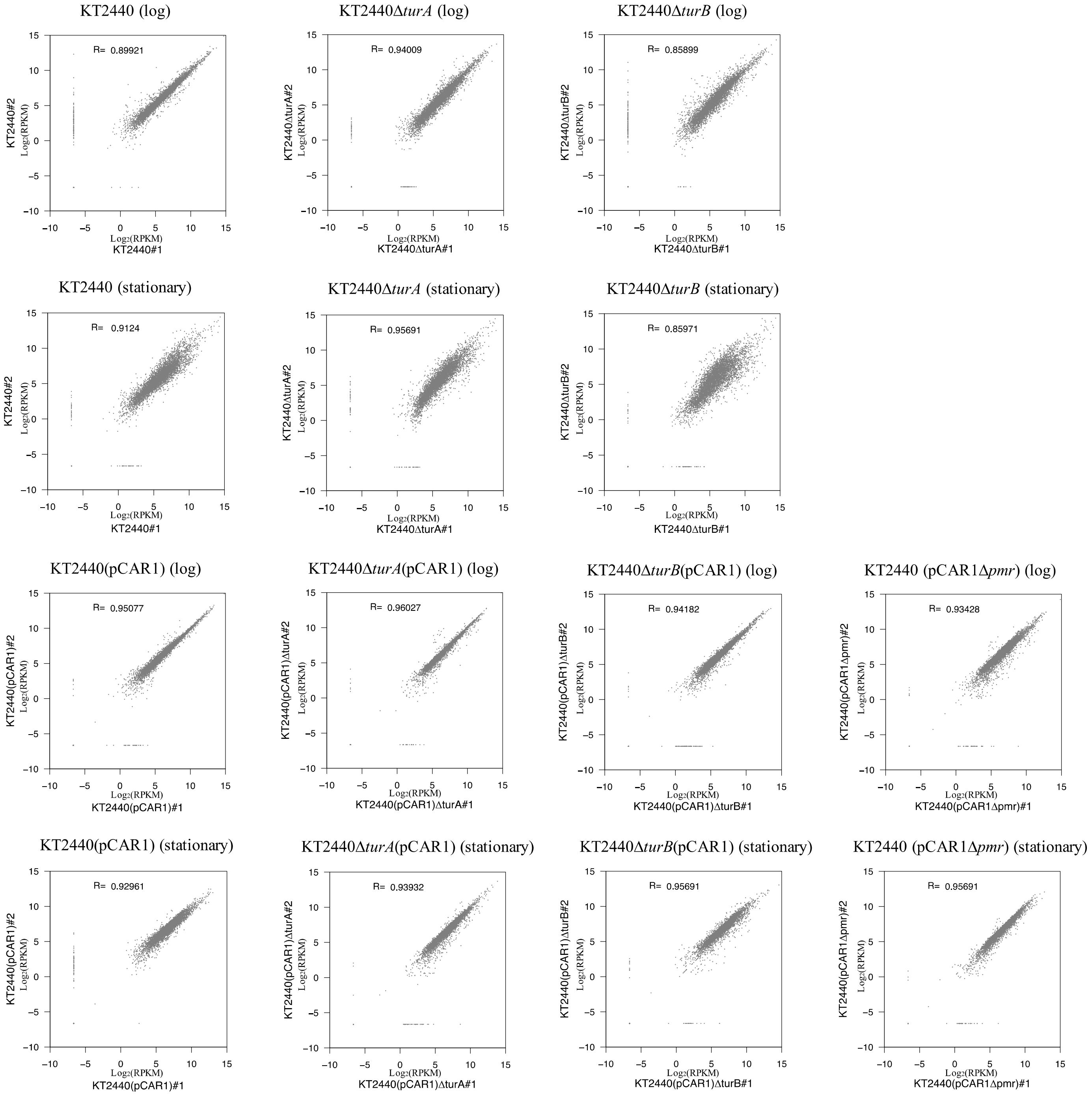

Supplementary Figure S2. Correlation between RPKM values of the two biological replicates for each strain. The number of mapped reads of each sample were normalized by RPKM (reads per kilobase of transcript per million mapped reads). Addition of 0.01 to all RPKM values was done to compensate for the variance of lowly expressed genes and to enable further calculation process. All RPKM values were transformed into log_2(RPKM) values. Scatter plots were made based on log_2(RPKM) values to estimate the correlation between transcriptomic profiles of each pair of biological replicates. Pearson’s correlation coefficients (R) are shown.

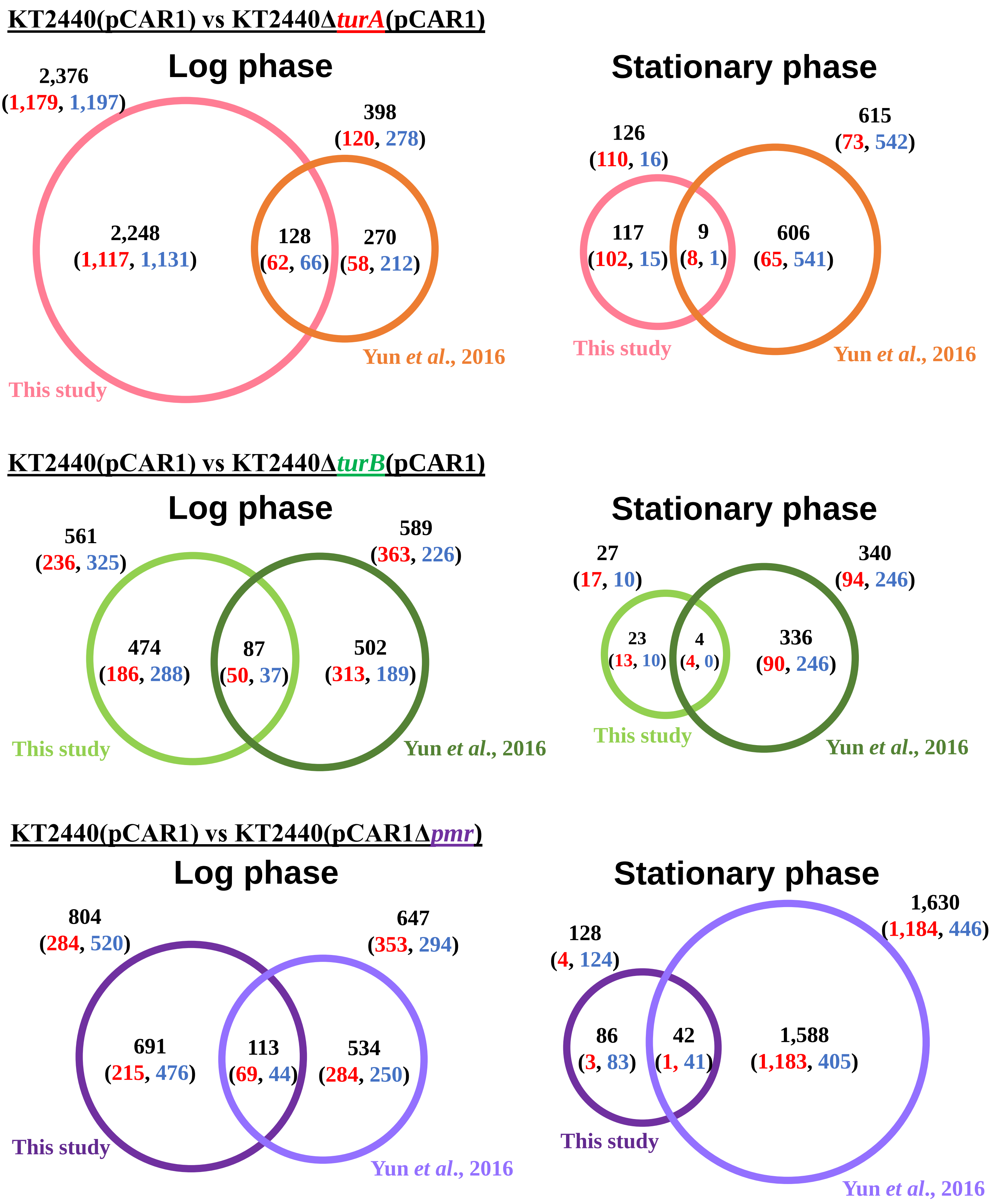

Supplementary Figure S3. Venn diagram of the differentially transcribed genes on the chromosome of KT2440Δ*turA*(pCAR1), KT2440Δ*turB*(pCAR1), and KT2440(pCAR1Δ*pmr*) in this study and those in the previous study (Yun et al. 2016). The numbers of differentially transcribed genes on the chromosome of KT2440Δ*turA*(pCAR1), KT2440Δ*turB*(pCAR1), and KT2440(pCAR1Δ*pmr*) compared with the wild-type KT2440(pCAR1) in this study are shown by pink, light green, and dark purple circles, respectively. Those in Yun et al. 2016 are shown by orange, dark green, and light purple circles, respectively. The numbers of up- and down-regulated genes are shown in red and blue digits, respectively.
